# Hepatic metabolic effects of Curcuma longa extract supplement in high-fructose and saturated fat fed rats

**DOI:** 10.1038/s41598-017-06220-0

**Published:** 2017-07-19

**Authors:** Fabrice Tranchida, Zo Rakotoniaina, Laetitia Shintu, Léopold Tchiakpe, Valérie Deyris, Mehdi Yemloul, Pierre Stocker, Nicolas Vidal, Odile Rimet, Abel Hiol, Stefano Caldarelli

**Affiliations:** 1 Aix Marseille Université, Centrale Marseille CNRS, iSm2 UMR 7313, 13397 Marseille, France; 2 Aix-Marseille Université, Laboratoire de Nutrition-Dietétique, Faculté de Pharmacie, Marseille, France; 3 Aix-Marseille Université, CNRS, Institut de Chimie Radicalaire UMR 7273, Equipe Sondes Moléculaires en Biologie et Stress Oxydant, Marseille, France; 4 SARL YELEN, Ensuès la Redonne, France; 5 Centre de coopération internationale en recherche agronomique pour le développement (CIRAD), UMR QualiSud, Université de La Réunion, Ecole Supérieure d’Ingénieurs Réunion Océan Indien (ESIROI), Saint Denis, France

## Abstract

The metabolic effects of an oral supplementation with a *Curcuma longa* extract, at a dose nutritionally relevant with common human use, on hepatic metabolism in rats fed a high fructose and saturated fatty acid (HFS) diet was evaluated. High-resolution magic-angle spinning NMR and GC/MS in combination with multivariate analysis have been employed to characterize the NMR metabolite profiles and fatty acid composition of liver tissue respectively. The results showed a clear discrimination between HFS groups and controls involving metabolites such as glucose, glycogen, amino acids, acetate, choline, lysophosphatidylcholine, phosphatidylethanolamine, and β-hydroxybutyrate as well as an increase of MUFAs and a decrease of n-6 and n-3 PUFAs. Although the administration of CL did not counteract deleterious effects of the HFS diet, some metabolites, namely some n-6 PUFA and n-3 PUFA, and betaine were found to increase significantly in liver samples from rats having received extract of curcuma compared to those fed the HFS diet alone. This result suggests that curcuminoids may affect the transmethylation pathway and/or osmotic regulation. CL extract supplementation in rats appears to increase some of the natural defences preventing the development of fatty liver by acting on the choline metabolism to increase fat export from the liver.

## Introduction

Native to Southeast Asia, *Curcuma longa* (CL) also known as turmeric is one of the most studied natural products. It is used as a culinary spice, as well as in traditional medicine for the treatment of many diseases^1^. In animal models, curcumin, the main curcuminoids in CL may be lipotropic, i.e., capable to hasten the removal of fat from liver and/or reduce hepatic lipid synthesis or deposits leading to reducing or preventing hepatic steatosis development^2, 3^. Previous studies have shown that curcumin may prevent fatty liver disease through multiple mechanisms including modulations of some antioxidative and anti-inflammatory properties^4^, signal transduction^5^, specific transcription factors activity^4^ and the expression of genes involved in lipid homeostasis^6^. The relevance of these mechanisms is not yet completely clear and the hypolipidemic action of curcumin may be conditioned by the pathway or condition that developed the fatty liver disease, the concentration of curcumin, the duration of curcumin addition, and the animal model used. However, the cited studies usually were performed using high concentration of curcuminoids, at doses not compatible with human use. Recently, a possible beneficial effect of a lower dose of curcuminoids in rats fed with a high fructose and saturated fatty acid (HFS) diet was observed in a NMR metabolomics and GC-MS lipidomics study of the serum^7^. The HFS diet largely mimics that of Western societies^8^, leading to metabolic abnormalities such as insulin resistance (IR) on rats^9^. Moreover, fructose consumption is suspected to promote the development of nonalcoholic fatty liver disease (NAFLD)^10^.

NMR spectroscopy has long been proven as an analytical tool of choice for untargeted metabolomics^11^, due to its capability at identifying tens of metabolites with minimal sample manipulation. More specifically, high-resolution magic-angle spinning (HRMAS) ^1^H NMR spectroscopy is a non-destructive analytical technique that provides well-resolved spectra of mobile species in tissue without the need for extraction^12^. This is achieved by spinning of the sample around inside the NMR probe at an angle of 54.7 ° with respect to the magnetic field at rotational frequency of a few kHz. In the case of fragile samples that could be damaged by rotation stress, protocols of sample handling have been described allowing for much slower spinning^13, 14^. HRMAS NMR has been successfully used to discover metabolic biomarkers to study in a variety of fields, notable examples being nutrition models and cancer discrimination^15, 16^. Furthermore, NMR-based metabolomics on liver extracts has provided useful information about the mechanisms involved in the development of metabolic alterations caused by high-fat and/or high-carbohydrate diets in rodents^17, 18^. Gas chromatography-mass spectroscopic (GC-MS) is a well-established technique for the analysis of thermally stable compounds, as it combines a high separation efficiency with selective and sensitive mass detection, which enable the rapid identification of metabolite profiles of biological samples using large MS databases^19^. GC-MS has been for many years one of the techniques of choice for the profiling of fatty acids. Consequently, the application of GC-MS in metabolomics/lipidomics research has developed considerably in various fields of research, such as microbiology, plant and medical sciences^20^, for instance.

In this work, a metabolomics approach based on HRMAS ^1^H NMR spectroscopy and GC-MS lipidomics was performed to highlight metabolic variations of liver tissue of rats fed a HFS diet with the addition of a Curcuma longa extract (1% in curcuminoids). To the best of our knowledge, this is the first study on this topic using this specific analytical approach. Glutathione (GSH) and lipid peroxidation were also measured in order to assess the oxidative stress level in each liver sample.

## Results

### Biochemical analysis

No significant difference was observed in liver triglycerides (Table 1). GSH and MDA assay were performed to appreciate the antioxidant capacity and lipid peroxidation level of the liver. Our results showed an increase of the MDA concentration (66 and 42% respectively) in HFS and HFS + C groups associated with a trend for higher tissue GSH, GSSG and total glutathione (TGlu) levels (Table 1). Results of body weight gain and serum biochemical analysis previously reported for this model^7^ are given for the sake of comparison in Supplementary Table S1.

**Table 1:** Triglycerides, MDA and glutathion concentrations in liver samples.

Group	Controls	HFS	HFS + C
Triglycerides liver (µmol/g)	8.76 ± 1.25	7.35 ± 1.16	7.86 ± 0.65
MDA (µmol/mg)	0.0038 ± 0.001	0.0063 ± 0.0013*	0.0054 ± 0.0012*
GSH (µmol/g)	0.20 ± 0.043	0.42 ± 0.096	0.38 ± 0.093
GSSG (µmol/g)	1.07 ± 0.14	1.30 ± 0.22	1.66 ± 0.18
TGlu (µmol/g)	1.27 ± 0.11	1.72 ± 0.28	2.04 ± 0.17*

HFS high fructose and saturated fatty acids, C curcuma (administration of hydroalcoholic extract of tumeric 100 mg/kg/day). MDA Malondialdehyde; TGlu: Total glutathione (GSH + GSSG); GSH: reduced glutathione; GSSG: oxidized glutathione. Values are mean ± S.E.M (n = 6–12 rats/group). *P < 0.05 vs. the control.

### Liver fatty acid (FA) profile

In all experimental groups, the major liver FA were: palmitic (16:0), stearic (18:0), oleic (18:1n-9), cis-vaccenic (18:1n-7), linoleic (18:2n-6), arachidonic (20:4n-6) and docosahexaenoic acids (22:6n-3 or DHA) (Supplementary Table S2). In general, the change in liver FA composition between the controls and the HFS group was similar to that previously described^7, 21^ for serum. In short, a significant decrease of polyunsaturated fatty acids (PUFA, −27%) was observed in the HFS group while monounsatured fatty acids (MUFA) were increased (2 fold). Among the PUFA, linoleic acid (18:2n-6) for n-6 PUFA, eicosopentaenoic acid (20:5n-3 or EPA) and alpha linolenic (C18:3n-3) acids for n-3 PUFA were species with the highest relative decrease. The addition of the extract of curcuma to the HFS diet did not modify these tendencies. However, the comparison between HFS and HFS + C groups showed a significant decrease of palmitic (C16:0), palmitoleic (C16:1n-7) and linoleic acid (18:2n-6) (−17%, −35% and −14%, respectively), associated with a relevant increase of gamma linolenic (C18:3n-6, more than 4 fold), arachidic (C20:0, 8 fold), gondoic (C20:1n-9, 3 fold), arachidonic (C20:4n-6, +14%) and docosapentaenoic (C22:5n-6, more than 4 fold) acids.

### ^1^H NMR spectroscopy of liver samples

**Figure 1:**
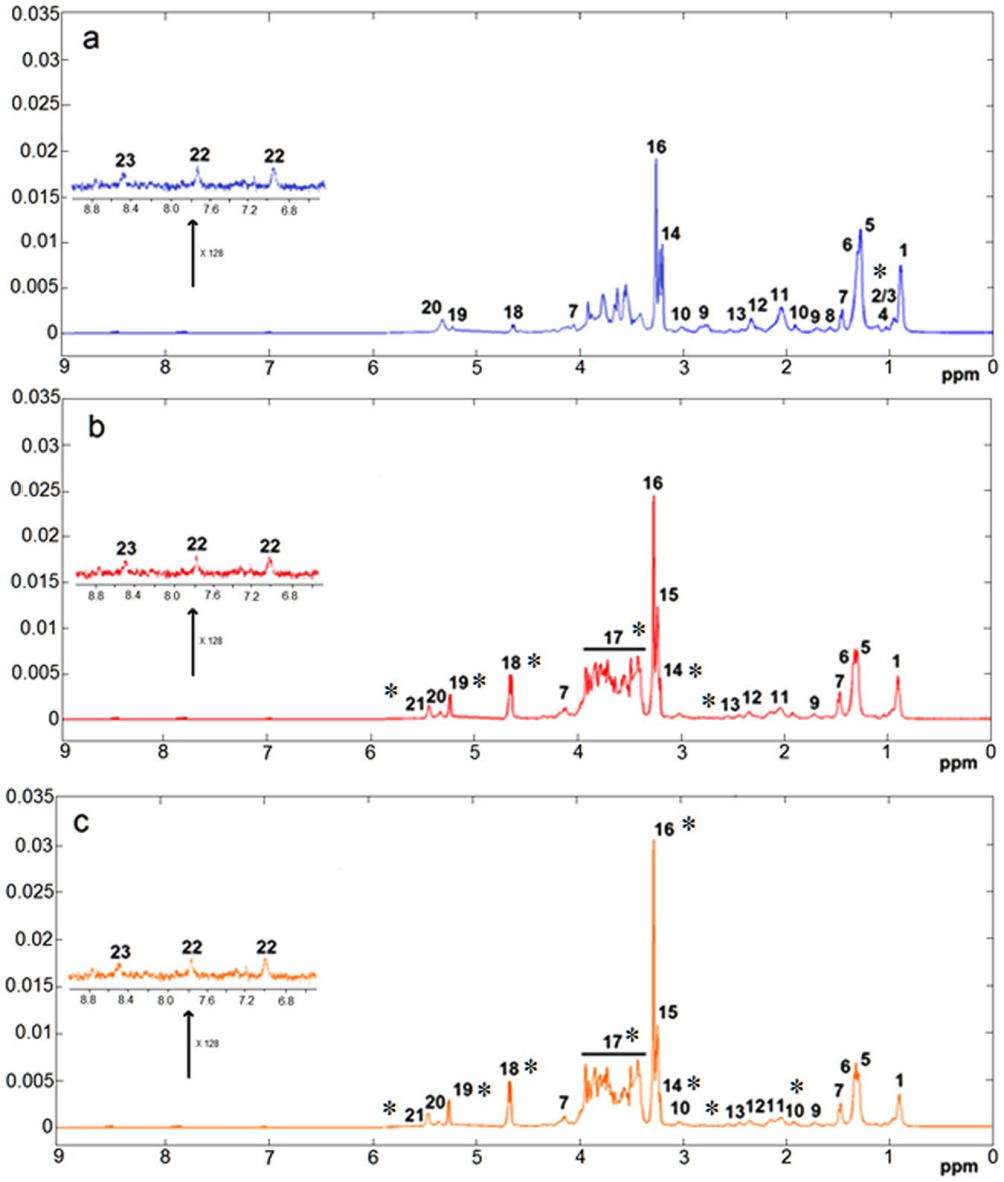
Average spectra of liver from ^1^H HRMAS CPMG experiment. (**a**) Control group, (**b**) HFS group, and (**c**) HFS + C group. Assignments: 1, lipids; 2, isoleucine; 3, leucine; 4, valine; 5, lipids; 6, lactate; 7, alanine; 8, lipids; 9, lysine; 10, acetate; 11, glycoproteins (acetyl); 12, glutamine; 13, glutamate; 14, choline; 15, phosphocholine/glycerophosphocholine (PC/GPC); 16, betaine; 17, alpha-glucose and beta-glucose/glycogen; 18, beta-glucose; 19, alpha-glucose; 20, lipids; 21, glycogen; 22, histidine; 23, ATP/ADP/AMP. *Denotes the main changes.

Figure 1(a,b and c) shows the average HRMAS ^1^H NMR spectra of all groups. Full assignments of the metabolites signals using 2D NMR experiments are given in Supplementary Table S3. Visual inspection on the average spectra showed already clear differences between control and HFS groups. The spectra of liver from HFS groups are dominated by the signals of glucose, glycogen, betaine, PC/GPC lipids and lactate correlated with the hyperglycemia of the rat under high fructose and fat diet. Furthermore, rats supplemented with extract of curcuma had on average higher level of betaine compared to HFS group, the signal of which (singlet at 3.27 ppm) was quantified across the groups (Table 2). The concentration of betaine in rat liver was in the micromoles per gram range, in agreement known reported homeostatic concentrations^22^. To gain further insight into the related metabolic pathways, the betaine precursor choline was also quantified using the singlet at 3.19 ppm.

**Table 2:** Betaine and choline concentrations in liver samples.

Group	Controls	HFS	HFS + C
**Betaine** (µmole/g of tissue)	6.42 ± 1.18	8.21 ± 0.95	12.73 ± 1.67*
**Choline** (µmole/g of tissue)	8.79 ± 1.02	3.63 ± 0.26**	3.23 ± 0.39**

Values are mean ± S.E.M (n = 6–12 rats/group). *P < 0.05 vs. the control and the HFS group, **P < 0.01 vs. the control.

### Multivariate statistical analysis of liver samples

**Figure 2:**
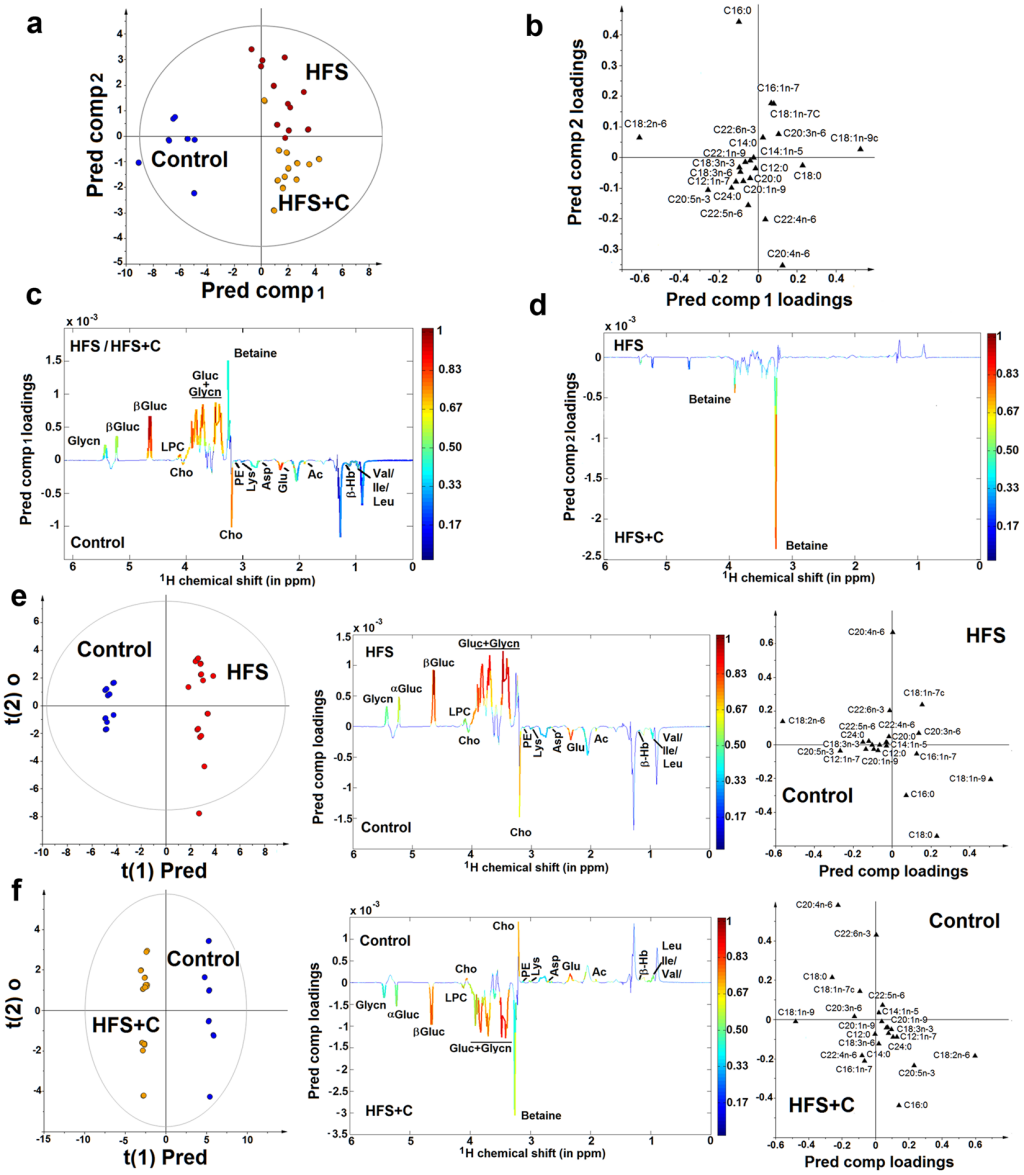
OPLS-DA score and loadings plots. (**a**) OPLS-DA score plot for Control, HFS and HFS + C groups. (**b**,**c**,**d**) OPLS-DA loadings plot representing the weights of the relative fatty acid contents and the NMR signals, respectively, along the two predictive components derived from OPLS-DA model of liver samples obtained from controls, HFS and HFS + C groups. OPLS-DA score and loadings plots representing the weights of the NMR signals and the relative fatty acid contents, along the predictive component derived from OPLS-DA models of liver samples obtained from (**e**) control and HFS groups, (**f**) control and HFS + C groups. The positive or negative phase of the resonance signals represents the relative concentration variation of metabolites derived from the covariance matrix. Signals from NMR data are color-coded according to their weights related to the correlation between the X and Y matrices, with red corresponding to highly correlated (r > 0.8) and blue (r < 0.2) indicating no correlation with sample class. Metabolites significantly discriminant were annotated on the model coefficient plot. Abbreviations: Ac = acetate, Asp = aspartic acid, β-Hb = β-hydroxybutyrate, Cho = choline, Glu = glutamate, Gluc = Glucose, Glycn = glycogene, Ile = Isoleucine, Leu = leucine, LPC = Lysophosphatidylcholine, Lys = lysine, PE = Phosphatidylethanolamine, Val = Valine.

OPLS-DA was performed for all three groups using both NMR and GC-MS datasets. The resulting scores plot (built with 2 predictive components, Fig. 2a) showed a clear discrimination between the three studied groups with a p-value of 3.35 10^−20^ (R2Y = 0.72, Q2Y = 0.64), using 63% of the total X-variance. The loading plots illustrate the contribution of the signal from several metabolites to the discrimination model. The relative relevance in the OPLS-DA of the potential biomarkers is listed in Table 3. The first predictive component was related to the diet-induced metabolic variations involving a decrease of several aminoacids (aspartate, glutamate, isoleucine, leucine, lysine, valine), acetate, β-hydroxybutyrate, phosphoethanolamine, choline, lauric (C12:0), lauroleic (C12:1n-7), alpha-linoleic (18:2n-6), alpha-linolenic (C18:3n-3), gamma linolenic (C18:3n-6), arachidic (C20:0), gondoic (C20:1n-9), eicosopentaenoic (C20:5n-3), adrenic (C22:4n-6), erucic (C22:1n-9), docosapentaenoic (C22:5n-6), lignoceric (C24:0) acids associated with an increase of betaine (relevant for HFS + C group), glucose, glycogen, lysophosphatidylcholine, palmitoleic (C16:1n-7), steric (C18:0), oleic (C18:1n-9), cis-vaccenic (C18:1n-7), dihomo-gamma-linolenic (C20:3n-6) and arachidonic (C20:4n-6) acids in HFS/HFS + C groups (Fig. 2b and c). The second predictive component reflected the curcuma-induced metabolic variations involving a decrease of palmitic (16:0), palmitoleic (C16:1n-7), cis-vaccenic (18:1n-7) and alpha-linoleic (18:2n-6) acid combined with an increase of betaine, lauric (C12:0), lauroleic (C12:1n-7), gamma linolenic (C18:3n-6), arachidic (C20:0), gondoic (C20:1n-9), arachidonic (C20:4n-6), eicosopentaenoic (C20:5n-3), adrenic (C22:4n-6), docosapentaenoic (C22:5n-6) and lignoceric (C24:0) acids (Fig. 2b and d).

**Table 3:** Significantly differential metabolites in the rat liver of control, HFS and HFS + C group.

	HFS/Controls^a^	P-value	Rank^b^	HFS + C/Controls^a^	P-value	Rank^b^	HFS + C/HFS^a^	P-value	Rank^b^
***Metabolites no Fatty acids***									
Acetate	0.71	<0.01	6	0.64	<0.00001	5			
Aspartate	0.66	<0.01	8	0.61	<0.01	10			
β-hydroxybutyrate	0.55	<0.05	10	0.49	<0.05	14			
Betaine				1.58	<0.01	7	1.35	<0.05	1
Choline	0.43	<0.01	4	0.36	<0.001	4			
Phosphatidylethanolamine	0.82	<0.05	11	0.74	<0.01	12			
Glucose	4.50	<0.00001	1	4.85	<0.00001	1			
Glycogen	12.87	<0.001	5	15.64	<0.001	6			
Glutamate	0.48	<0.00001	3	0.44	<0.00001	3			
Isoleucine	0.60	<0.01	7	0.57	<0.01	13			
Lysophosphatidylcholine	1.58	<0.0001	2	1.60	<0.00001	2			
Leucine	0.53	<0.01	12	0.52	<0.01	9			
Lysine	0.79	<0.05	9	0.75	<0.001	11			
Valine	0.64	<0.05	7	0.61	<0.01	8			
*Fatty acids*									
C12:0 Lauric acid							N/A	<0.05	12
C12:1n-7 Lauroleic	0	<0.0001	3	0.22	<0.001	6	N/A	<0.01	9
C16:0 Palmitic acid							0.82	<0.01	1
C16:1n-7 Palmitoleic acid	2.31	<0.05	11				0.68	<0.001	7
C18:0 Steric acid	1.24	<0.05	15	1.26	<0.05	10			
C18:1n-9 Oleic acid	2.48	<0.0001	4	2.53	<0.001	3			
C18:1n-7 Vaccenic acid	1.51	<0.01	13	1.25	<0.05	14	0.89	<0.05	6
C18:2n-6 Linoleic acid	0.53	<1E-11	1	0.45	<1E-17	1	0.84	<0.05	3
C18:3n-3 alpha Linolenic acid	0.06	<1E-6	2	0.09	<0.0001	2			
C18:3n-6 gamma Linolenic acid	0.09	<0.0001	5	0.32	<0.01	8	3.60	<0.05	11
C20:0 Arachidic acid	0.05	<0.05	12	0.35	<0.05	13	7.15	<0.05	14
C20:1n-9 Gondoic acid	0.16	<0.01	9	0.45	<0.05	9	2.78	<0.05	10
C20:3n-6 Dihomo gamma linolenic acid	2.93	<0.01	8	2.55	<0.001	4			
C20:4n-6 Arachidonic acid				1.17	<0.05	11	1.15	<0.05	2
C20:5n-3 Eicosopentaenoic acid	0.12	<0.00001	6	0.22	<0.001	5	1.83	<0.01	8
C22:1n-9 Erucic acid	0	<0.05	14	0	<0.05	12			
C22:4n-6 Adrenic acid							4.88	<0.05	5
C22:5n-6 Docosapentaenoic acid	0.13	<0.01	10				5.82	<0.01	4
C24:0 Lignoceric acid	0.13	<0.001	7	0.24	<0.001	7	1.91	<0.01	13

The liver metabolites that contributed significantly to the discrimination between the different diets in the OPLS-DA models from the ^1^H NMR data and GC/MS data. ^a^Fold change in metabolite level followed by P-value and ^b^Rank in variable importance projection (VIP). N/A not applicable.

To better identify the significant liver metabolic differences between three different groups, pairwise comparative OPLS-DA were conducted with one orthogonal and one predictive component calculated for each of the models. The results are also displayed in the forms of scores plots and corresponding loadings plots of the predictive latent variable (Fig. 2e and f). The OPLS-DA scores plot showed a clear discrimination along the predictive component between the controls and the rats that received a diet enriched in fructose and saturated fat (p-value of 3.60 10^−17^, R2Y = 0.99, Q2Y = 0.97 for control vs HFS group; p-value of 2.86 10^−23^, R2Y = 0.99, Q2Y = 0.98 for control vs HFS + C group). The corresponding loading plot was almost identical to the one obtained above from the OPLS-DA model performed on the three groups, except that betaine and arachidonic (C20:4n-6) acid increased significantly only in the HFS + C group, whereas palmitoleic (C16:1n-7) and docosapentaenoic (C22:5n-6) acids, respectively increased and decreased only in the HFS group. Furthermore, the OPLS-DA score and loading plots of the discrimination of the HFS and HFS + C groups (Supplementary Fig. S1) (p-value of 3.04 10^–6^, R2Y = 0.90, Q2Y = 0.78) confirmed the curcuma-induced metabolic variations described above (Fig. 2b and d). The statistical model was tested for robustness by a Y-permutation performed on PLS-DA, which confirmed the observed metabolic variations could not be ascribed to random effects (Supplementary Figs S2 and S3).

## Discussion

### Effects of HFS and HSF + C diets on the metabolism

#### Biochemical tests

Consumption of a HFS diet leads to IR for this model, with a tenfold increase in the HOMA-IR in the serum (Table S1). Although, a two-threefold increase of the serum TG concentration was observed in HFS groups compared to control^7^, no significant difference could be detected in this study in the hepatic TG level among the groups. Further studies will be required to elucidate the difference in the homeostatic states in liver and serum, associated to increased excretion of TG from the liver. It is likely a combination of increased VLDL-TG production, decreased clearance and hepatic inflammation that contribute to the hypertriglyceridemia induced by a HFS diet^23, 24^.

Rats from both HFS groups had increased lipid peroxidation and a trend for higher tissue GSH and GSSG levels (Table 1), suggesting an increase in the production of reactive oxygen species (ROS). These results are in accordance with a previous study showing that an elevated hepatic antioxidant capacity can be induced by a carbohydrate-rich diet in rat livers^25^. Thus, and in contrast to previous results on serum^7^, livers of rats fed a HFS diet showed an increased capacity to resist against oxidative stress probably by an adaptive response in the early stages of IR.

The oral administration of curcuma extract did not correspond to a reduction of the lipid peroxidation induced by the HFS diet, in contrast to previous observations that curcumin in the CL powder could eliminate reactive oxygen species^26^. This discrepancy could be explained by the lower dose of curcuminoids and the composition of the diet used here. Nonetheless, HFS + C group shows a significantly higher GSSG and level of TGlu, suggesting an active conversion of GSH to GSSG by glutathione peroxidase (GPx) and an increase in γ-glutamylcysteine synthetase (γ-GCS) activity, which catalyzes the rate limiting step in GSH biosynthesis. This is in agreement with a previous observation that curcuminoids could increase γ-GCS and GPx expression as a defense mechanism against oxidative stress^27^.

### Metabolic NMR and GC/MS variations between Control and HFS/HFS + C groups

#### Fatty acids (FA) metabolism

As previously observed^28, 29^, the liver FA profile in rats fed a HFS regime was characterized by a higher proportion of MUFA and by a lower proportion of PUFA (supplementary information Table S2), while the SFA remained approximately constant. Consequently, the hepatic PUFA/MUFA ratio, which is sometimes used as a surrogate measure of lipid peroxidation^30^, was significantly lower in HFS rats compared to the controls. This indicates increased lipid peroxidation as corroborated by the high levels of MDA detected in HFS groups. Most of this change can be ascribed to a more than two fold positive change of oleic (C18:1n-9) acid, in conjunction with a almost compensating decrease in linoleic (18:2n-6) acid. Numerous investigations have shown an enhancement of MUFA production in fructose and/or saturated fat administration, due to an increase in hepatic Δ9D^31, 32^. The decrease of n-3 PUFA and the defect in Δ6D and Δ5D activity induced by the HFS diet could be involved in the early stages leading to IR^33^.

#### Glucose metabolism

HFS rats significant increase in carbohydrate metabolites, with more then 10 times higher levels of glycogen and fivefold increase of glucose, suggesting an activation of glycogenesis and gluconeogenesis. Both molecules were identified by the statistical model as being the most discriminant biomarkers found via NMR metabolomics (Table 3). Since glucose was not part of the diet, the substrate for glycogen synthesis likely came from dietary fructose through the gluconeogenic pathway. Indeed, it has been observed that in fasted conditions the gluconeogenesis and glucose production from fructose were active processes^34^. Furthermore, fructose administration increases hepatic glycogen levels even more than an equivalent dose of glucose in both rats^35^ and humans^36^. The production of glycogen appears to result from both activation of glycogen synthase^37^ and inhibition of glycogen phosphorylase^38^ by the fructose-1-phosphate accumulated after administration of fructose^39^.

#### Aminoacids (AA) metabolism

Consistently with the observations of NMR metabolomics of the blood serum of this animal model^7^, a lower relative concentration of several aminoacids (aspartate, glutamate, lysine, the branched chain amino acids (BCAA) isoleucine, leucine and valine) was observed in both HFS groups compared to controls. All these amino acids are involved in the gluconeogenic and/or ketogenic metabolism in the liver, and thus it is not surprising to observe a reduction in their concentration in glucose- and glycogen- rich rat livers. Further along these lines, the decreased concentration of β-hydroxybutyrate indicates that beta-oxidation and ketogenesis were indeed reduced. On the other hand, hepatic glutamate has a more complex role, being at the crossing of several metabolic pathways such as maintenance of acid-base balance and as precursor of the most body’s important antioxidant glutathione^40^. The fact that this compound can be a biomarker of liver dysfunctions appears thus to be reasonable. Similarly, a decrease in glutamate level in liver may be related to the trend for to the higher concentration of tissue GSH to counter the oxidizing stress caused by a HFS diet.

#### Choline metabolism

Choline, phosphatidylethanolamine (PE) and lysophosphatidylcholine (LPC) were observed to be perturbed by the HFS diets, the first two metabolites being less (fold change of 0.43 and 0.82) and the last one more abundant (fold change of 1.58). In liver, choline is phosphorylated to phosphocholine in the CDP-choline pathway, which contributes about 70–80% of phosphatidylcholine (PC) formation. Alternatively, choline is oxidized to betaine, an important precursor of S-adenosylmethionine (SAM)^41^. PE is also involved in PC synthesis via the PEMT pathway^42^. Since PC is a required component of VLDL-TG^43^, the depletion of choline and PE in HFS groups is in accordance with the higher amount of VLDL-TG observed in the serum in association to this diet^5^. Incidentally, choline depletion could also point towards a possible role of the gut microbiota, which in high-fat diet-induced IR mice was found to reduce the biodisponibility of dietary choline by an increased conversion to methylamines leading to non-alcoholic fatty liver disease (NAFLD)^44^. In parallel to the above, the higher levels of LPC observed in HFS groups suggest an increased activity of phospholipase A2, which removes one of the fatty acid groups in PC.

#### Elevation of acetate

We have observed a higher level of acetate in HFS groups compared to the control group. Acetate stems from both intestinal microbial and endogenous production^45^. However, in this work, the oral vehicle contained 2% of ethanol, which is oxidized to acetate in the liver and on the other hand, it has been shown that fructose increases the rate of ethanol metabolism in both animals and man^46, 47^.

### Discriminant metabolites between HFS and HFS + C groups

The inspection of the OPLS-DA statistics, and more specifically of the loadings of the latent variables (Fig. 2b and d), show that a few key FAs and an increase of betaine were the most discriminant effects for the HFS + C and the HFS group.

#### Fatty acids

The main variations in FA composition between the two groups were provided by palmitic (C16:0) and linoleic (C18:2n-6) acids, more abundant in the HFS group, and the arachidonic (C20:4n-6) acid increased its relative concentration upon addition of Curcuma to the diet (Table 3). The decrease of C16:0 could be the result of increase of its incorporation into TG and away from pathways introducing to apoptosis^48^. Since arachidonic acid is biosynthesized from linoleic acid, the observed variation in relative concentrations of these two compounds seems to point towards an upregulation of the concerned enzymes, Δ6 and Δ5 desaturases. Furthermore, it has been observed that curcumin anti-inflammatory properties inhibited n-6 eicosanoid biosynthesis from arachidonic acid^49^, which could increase the availability of this latter for n-6 PUFAs biosynthesis. On the other hand, deregulation of one or several of the many pathways involving fatty acids cannot be excluded.

#### Betaine

**Figure 3:**
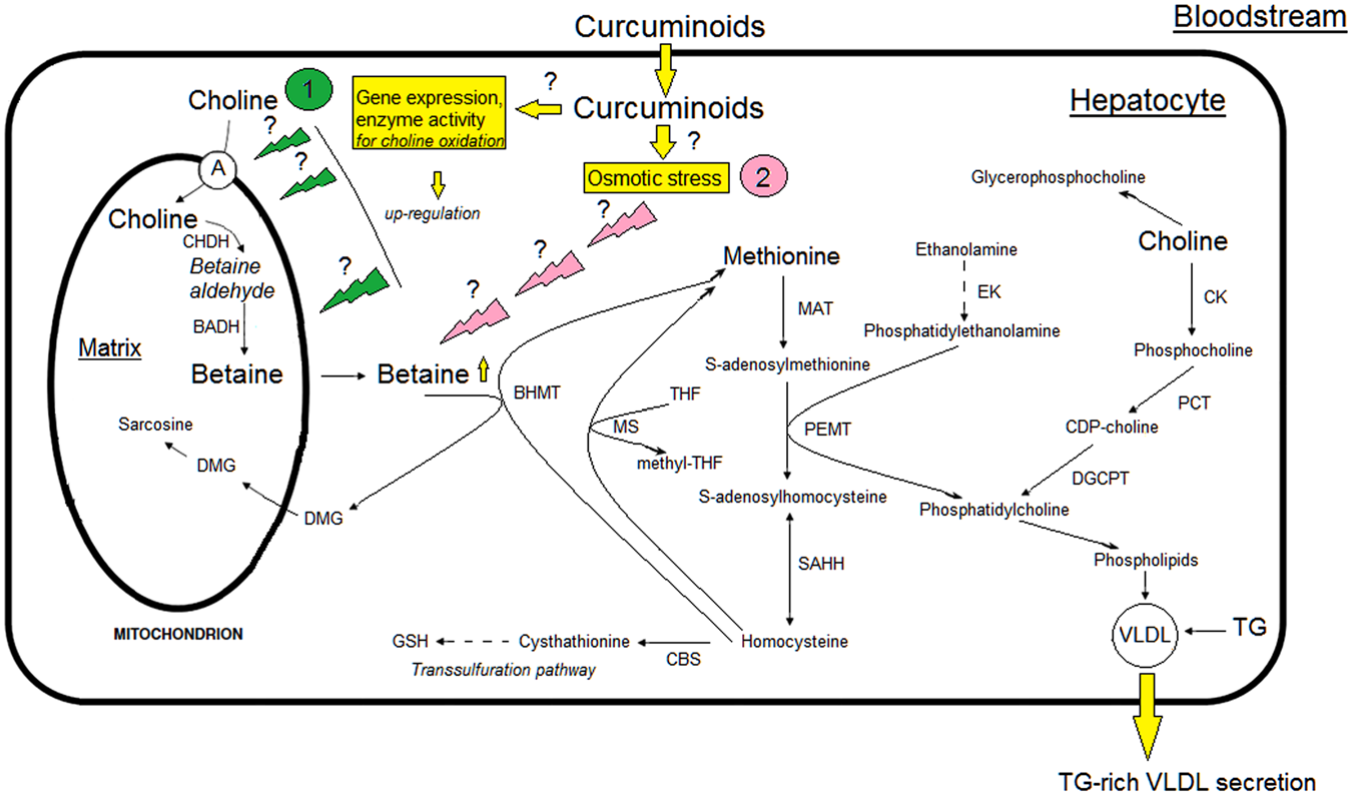
Overview of the metabolism of hepatic methylamine. (1) Choline oxidation pathway, the up regulation of gene expression and/or enzyme activity involved in this pathway. (2) Osmotic stress pathway by which curcuminoids might downregulate the BHMT enzyme activity in liver leading to high level of betaine. Choline transporter (A); choline dehydrogenase (CHDH); betaine aldehyde dehydrogenase (BADH); betaine homocysteine methyltransferase (BHMT); cystathionine β-synthase (CBS); choline kinase (CK); 1,2 diacylglycerol choline phosphotransferase (DGCPT); dimethylglycine (DMG); ethanolamine kinase (EK); methionine adenosyltransferase (MAT); methionine synthase (MS); phosphocholine cytidylyltransferase (PCT); phosphoethanolamine N-methyltransferase (PEMT); S-adenosylhomocysteine hydrolase (SAHH); tetrahydrofolate (THF).

The increase in betaine levels is perhaps the most striking difference emerging in the statistical differentiation of HFS rats when fed with the turmeric supplement. Curcuma can contain up to a few dozen micromolar concentrations of betaine^50^, which is insignificant compared to the levels of betaine found in this study, and can thus be neglected. The metabolism of betaine in the rat liver as a catabolic product of choline in and out of the mitochondria is represented in Fig. 3. In short, betaine, produced from choline, is a lipotrope and an important osmolyte which plays a key role in one of the pathways for methionine recycling in the liver and regeneration of S-adenosylmethionine (SAM) from homocysteine via betaine homocysteine methyltransferase (BHMT)^51^. Thus, the lipotropic effect of betaine is mainly related to the ability to transfer its labile methyl groups via the transmethylation pathway leading, among others, to phosphatidylcholine (PC) which is indispensable for the packaging and export of triglycerides in very low density lipoprotein (VLDL) from the liver (Fig. 3)^52, 53^. Interestingly, betaine supplementation in rats fed an ethanol or control diet increased hepatic VLDL secretion^54^ and may correct VLDL secretion inhibition initiated by choline deficiency^55^. Thus, studies should be conducted to determine whether curcumin and/or curcuminoids could modulate the activity of specific transcription factors that regulate the expression of genes involved in choline oxidation (choline transporter, choline dehydrogenase, betaine aldehyde dehydrogenase) or interact directly with these enzymes or transporter (Fig. 3).

A precise view of a possible perturbation of the metabolic pathways involving betaine regulation could not be described here, as dimethyglycine and methionine could not be identified in the ^1^H NMR spectra. The former molecule was not detectable at all in the NMR spectra of liver due to its lower average concentration in the liver tissue, about three orders of magnitude less than betaine^56^. Methionine was clearly identified only in 2D NMR spectra, since its signal was not resolved in the 1D ^1^H NMR spectra due to overlap with and blanking by glutamate and glutamine signals, which prevented its use in the statistical analysis.

Analysis of the literature does confirm a role of betaine in diet-induced metabolic diseases, but not in a conclusive way. For instance, by metabolomics approaches lower levels of betaine in the liver of obese Zucker rats or from high-fat diet induced obese mice^17, 18, 57^ were observed, while another study revealed an opposite effect in rats fed a high-fat diet^58^. In humans, its use in the treatment of NAFLD has been shown to lead to significant improvements of liver functions without adverse effects and may protect against worsening steatosis^59^.

It should also be noted that the expression of BHMT is highly sensitive to osmotic conditions^60, 61^. Indeed, it has been demonstrated in rat hepatoma cells that BHMT mRNA and protein expression as well as BHMT enzyme activity are downregulated under hyperosmotic conditions, which causes an increase of the intracellular concentration of betaine^60^. To this respect, a curcumin treatment was shown to induce hyperosmotic stress in yeast cells^62^. In that study, this effect was countered by a variation in glycerol, another osmotic agent. This metabolite could not be resolved in our statistical study, as its signals in the ^1^H NMR spectrum are buried below the glucose and glycogen resonances. Therefore, in inducing an osmotic stress, curcumin might downregulate the BHMT enzyme activity in liver (Fig. 3).

In conclusion, this work revealed a strong impact of a HFS diet, either in the presence or absence of Curcuma Longa, on the liver metabolite profile affecting glucose/glycogen, choline, FA and aminoacids concentrations. The diet induced insulin resistance. The dose of curcuminoids used here as a supplement of nutritional relevance was not sufficient to induce a protective reaction against the IR. However, a strong effect of CL extracts supplementation was observed on the content of liver betaine, which was found to increase by 35%. Thus, the transmethylation pathway and/or osmotic regulation may be impacted. The specific mechanism by which Curcuma increases the level of liver betaine remains to be determined and further studies are underway to clarify this issue.

## Methods

### Reagents

All chemicals used in this study were of the highest grade. Acetyl chloride, 2,6-di-tert-butylp-cresol, butylated hydroxytoluene (BHT), dansyl chloride, iodoacetic acid, methanol, hexane, the internal standard (23:0 methyl ester), beta-mercaptoethanol, potassium phosphate, betaine, curcumin and Supelco 37 component FAME (fatty acid methyl ester) mix were purchased from Sigma-Aldrich (Sigma-Aldrich Chimie S.a.r.l, Lyon, France).

### Preparation and characterization of hydroalcoholic extract of *Curcuma longa*

Powder of the rhizome of *C. longa* was provided by Laco SARL (Marseille, France). For the extraction, *C. longa* powder was macerated with hot water (80 °C) for 4 h, and the aqueous extract was evaporated under vacuum at 60 °C. The rhizome residue was re-extracted with ethanol at 60 °C during 2 h, filtered, and evaporated under vacuum. The final extract was a 1:1 mixture of the aqueous and alcohol extracts that was re-dissolved with 2% alcohol and then with 0.9% NaCl for the *per os* administration. The concentration of curcuminoids in the curcuma extract was determined by a UV-VIS spectrophotometer (Shimadzu). A 25 mg amount of sample extract was dissolved in 50 ml of ethanol by sonication. The sample was further filtered through 0.45 µm and diluted 1:25 in ethanol. The absorbance was measured at 426 nm and the curcuminoids were identified with curcumin pure standard with a molar absorbance of 1607 in ethanol. Total concentration of curcuminoids in the Curcuma extract was 1%. Curcuminoids composition of the extract was carried out by high-performance liquid chromatography (HPLC) with a Thermo Finnigan Spectra System UV6000LP (Thermo Fisher Scientific, Waltham, MA, USA) and a Pursuit XRS C18 column (250 × 4.6 mm and 5 µm, Agilent) with UV detection set at 426 nm. The sample (10 µl) was injected using the full-loop method. The separation of curcuminoids substances was achieved within 30 min using a mobile phase composed of HPLC-grade water containing 2% acetic acid (A) and acetonitrile (B) at 0.9 mL/min flow. The gradient elution was: 60% B (0–10 min), 90% B (10–30 min), the system returned to its initial condition (40% A/60% B) within 1 min, and was then equilibrated for 9 min. HPLC analysis revealed curcumin (58.8%), demethoxycurcumin (22.2%) and bisdemethoxycurcumin (19.0%). A HPLC chromatogram of this extract is shown in Supplementary Fig. S4. The antioxidant activity of the ethanol extract of *Curcuma Longa* has been attributed mainly to curcuminoid components present, as reported^63^. A profile of polar (methanol) and non-polar (hexane) extracts of Curcuma extract was performed by ^1^H NMR spectroscopy and Gas Chromatography/Electron Ionization Quadrupole Time of Flight Mass Spectrometry (GC/QTOF MS), respectively. ^1^H NMR spectroscopy identified mainly amino acids, organic acids and sugars. GC/QTOF MS analysis resulted in the detection of three peaks, these constituents were identified as ar-turmerone (49.3%), curlone (26.3%) and α-turmerone (24.4%) based on their mass fragmentation pattern and retention times. Detailed procedures for preparation of sample and instrumental analysis (Data S1 and S2), ^1^H NMR spectra and assignments of polar metabolites from the methanol extract (Fig. S5 and Table S4), GC/QTOF MS chromatographic profile and mass spectra of non-polar metabolites from the hexane extract (Figs S6 and S7) are provided in Supplementary information.

### Experimental animals and diets

As described previously^7^, thirty male Sprague-Dawley rats (180–200 g) purchased from Janvier (Le Genest-St. Isle, France) were housed in a temperature- and humidity-controlled condition with a 12 h light-dark cycle. After one week of adaptation under feeding (available *ad libitum*) with a standard diet (3.32 Kcal/g, SAFE, Augy, France), the rats were divided into three groups: a control group (n = 6) fed with the standard diet, a HFS group (n = 12) fed with a diet where the total carbohydrates and lipids present in the standard diet were replaced by 61.7% fructose and 12% lard, respectively (4.3 Kcal/g, SAFE, Augy, France) and a HFS + C group (n = 12) further supplemented with an hydroalcoholic extract of Curcuma at a dosage of 100 mg/kg body weight/day by oral gavage. This dosage contains 1 mg/kg per day of curcuminoids. According to a formula of conversion of animal doses to human doses provided by the Food and Drug Administration^64^ based on body surface area, the human equivalent dose would be approximately 0.16 mg/kg of curcuminoids in adult. It is noted that the joint report of the Food and Agriculture Organization and the World Health Organization on food additives^65^ recommends an intake of 1 mg/kg/day of curcuminoids in the population. After 10 weeks of treatment, the rats were fasted overnight, anesthetized, and blood sample was collected from the vena cava just prior to sacrifice. Samples were then centrifuged for 10 min at 1000 g and sera collected. Liver was removed, washed in ice-cold 0.9% saline to remove residual blood, weighed and immediately snap-frozen in liquid nitrogen and stored along with the serum at −80 °C until analysis. Animal care and experimental procedures were approved by the Animal Ethics Committee of the Faculty of Pharmacy of Aix-Marseille Université (Marseille, France) and in accordance with the guidelines of the French Ministry of Food and Agriculture. The composition of each diet is reported in Supplementary Table S5.

The experimental design was selected according the key principle of the economy of animal sacrifice. Thus, on the basis of numerous studies^66, 67^, the addition of a fourth group of rats being fed a control diet with the addition of just a Curcuma extract was not esteemed necessary. Indeed, no differences were observed between a control group and one having received turmeric extract or high dose of curcumin on blood glucose, glycosylated hemoglobin, plasma insulin, plasma leptin, lipid profiles (plasma, liver, kidney), liver weights, hepatic glycogen, hepatic glucose and lipid regulating enzyme activities, lipoprotein lipase activity, serum liver biomarkers and antioxidant defenses (liver lipid peroxidation, plasma total antioxidant status, liver antioxidant enzymes as superoxide dismutase, catalase, glutathione peroxidase)^68–71^.

### Biochemical analysis

The content in triglycerides of the liver tissue was determined using an enzymatic assay with a commercial kit according to the manufacturer’s instructions (BioVision Research Products, CA, USA).

Malondialdehyde (MDA) levels were measured by the thiobarbituric assay, and were taken as an index of lipid peroxidation in liver. The samples were processed using a procedure similar to that described for serum MDA determination^7^. Briefly, equal amounts of tissues were taken from livers and homogenized in 10 mM potassium phosphate buffer, pH 7, containing 1.4 mM beta-mercaptoethanol and filtered through gauze then centrifuged at 28,000 g for 45 min. The supernatant fraction was then used for MDA determination.

Reduced glutathione (GSH) and oxidized glutathione (GSSG) were determined using the method previously reported^72^. The liver homogenate supernatants (150 µl) were derivatized with 40 mM iodoacetic acid at pH 9.0 for 15 min. The pH was adjusted to 9.0 with KOH/tetraborate solution (150 µl). Dansyl chloride was added (10 mM final), and samples were left at room temperature for 24 h in the dark to form S-carboxymethyl-*N*-dansyl-GSH and *N,N*9 bisdansyl-GSSG adducts. Unreacted dansyl chloride was extracted with chloroform and the GSH and GSSG adducts were separated by HPLC (see below) and quantified relative to standards using a fluorescence detector (excitation wavelength, 335 nm, emission wavelength, 515 nm). HPLC analysis was performed using a Waters Alliance System (Waters SAS, Guyancourt, France) equipped with a Waters 2690 XE separation module and a Waters 474 Scanning fluorescence detector controlled by the Waters Millenium Chromatography manager software. Separation was achieved at room temperature on a 3-aminopropyl column (250 mm × 4.6 mm; 5 µm; Macherey-Nagel, Hoerdt, France) with an isocratic flow rate of 1.2 ml/min. Solvent A is a 0.2 M acetate buffer (pH 4.6) and solvent B is 80% (v/v) methanol/water. Quantification was based on peak area.

### Liver fatty acids (FA) determination by gas chromatography-mass spectrometry

Sample of liver tissue (100 mg) taken from the left lateral lobe were homogenized in 2 ml of methanol-chloroform 4:1 (v/v) using a Ultra-Turrax homogenizer (Sigma-Aldrich). BHT (50 µg BHT/ml) to prevent fatty acid oxidation and the internal standard (100 µg/ml) were dissolved in the methanol solution which was used for homogenization of the sample. For FAME synthesis, an effective acid catalyst acetyl chloride (200 μl) was slowly added to each homogenate and the samples were incubated in screw-capped glass tubes for 60 min at 100 °C in a water bath. After cooling to room temperature, 0.75 ml of hexane was added to the samples. After vortexing, the upper organic phase was collected. This step was repeated twice. The combined organic phases were evaporated to dryness under nitrogen and the lipids were resuspended in 100 μl of hexane prior to analysis. The GC-MS analysis were performed in duplicate on the extract. One microliter of each esterified sample was injected into a Thermo Scientific ITQ 700 (Thermo Fisher Scientific) gas chromatograph-ion trap spectroscopy equipped with PEG columns (30 m × 0.25 mm id., 0.25 μm thickness) (DB-FFAP Agilent Technologies, France) in duplicate. Helium was used as the carrier gas at a constant flow rate of 1.0 ml/min. The injection temperature was set to 260 °C and the split ratio of the injector was 1:50. The initial oven temperature was set to 140 °C. After 1 min the oven temperature was increased from 140 °C to 250 °C at a rate of 4 °C/min and was then maintained at 250 °C for 20 min. The transfer line temperature and ion source temperature were controlled at 260 °C and 250 °C respectively. Ionization was achieved using a 70 eV electron beam. Mass spectra were acquired from m/z 50 to 650 at a rate of 2 s in full scan mode. Data collection and processing were performed by means of XCALIBUR software, (version 2.0 Thermo Fisher Scientific). In order to monitor the reproducibility and stability of the GC/MS, equal aliquots of all liver homogenate samples were mixed together to prepare a pool of quality control (QC) samples. QC samples were injected (n = 5) intermittently through the analytical experiment after the initial equilibration. Principal component analysis (PCA) was carried out using SIMCA-P 13 (Umetrics, Umea, Sweden) to obtain an overview of FAs from the QCs and the study samples where tight clustering of the QCs in the PCA scores plot was observed (Supplementary Fig. S8), indicating good reproducibility of the data^73^.

Fatty acids identification was made by comparing retention time and mass spectra with a standard mixture of 37 fatty acid methyl esters. The relative amount of each fatty acid was determined by the area of the chromatographic band normalized by the total area. Estimation of desaturase activity of delta-9 desaturase (Δ9D), delta-6 desaturase (Δ6D) and delta-5 desaturase (Δ5D) was performed by the product/substrate (18:1n-9/18:0) and (16:1n-7/16:0) ratios, the (20:3n-6/18:2n-6) ratio, the (20:4n-6/20:3n-6) ratio respectively^74^.

### Sample preparation and NMR experiments

^*1*^ *H NMR Spectroscopy*. Samples of liver tissue taken from the left lateral lobe were prepared in duplicate, each approximately 15 mg, and placed into a 4 mm ZrO_2_ HRMAS rotor with a cylindrical insert. D_2_O (5 μl) was added to the samples to provide field-lock signal. All NMR experiments were carried out on a Bruker Avance spectrometer operating at 400 MHz for the ^1^H frequency equipped with a ^1^H/^13^C/^31^P HRMAS probe. Spectra were acquired at 278 K with a spin rate of 4 kHz. A Carr−Purcell−Meiboom−Gill (CPMG) NMR spin echo sequence with an overall spin echo time of 80 ms, preceded by a water presaturation pulse during a relaxation delay of 1.2 s ([presat-90°-(τ-180°-τ)_n_]), was employed to reduce signal intensities of lipids and macromolecules. For each spectrum, 256 free induction decays (FID) of 16 k data points were collected using a spectral width of 8000 Hz.

In addition, a pulse-acquire sequence with water signal presaturation during a relaxation delay of 1.2 s was recorded. Two hundred fifty-six transients and 16k data points were collected using a spectral width of 8000 Hz. For both sequences, the FIDs were multiplied by an exponential weighting function corresponding to a line broadening of 0.3 Hz and zero-filled once prior to Fourier transformation. Subsequently, the spectra were phased and baseline corrected manually and referenced to the alanine methyl signal (δ = 1.47 ppm). Assignments of the metabolite signals were performed using ^1^H−^1^H TOCSY^75^, ^1^H−^13^C HSQC^76^, ^1^H−^13^C HMBC^77^ spectra and using reference signals published in the literature^78, 79^.

Betaine was quantified by comparison to the spectrum of the pure compound. Curve fitting using TOPSPIN software (version 3.1, Bruker Biospin, Germany) was required to quantify the singlet due to the methyl protons of betaine, in order to minimize the effects of nearby peaks. Choline was quantified using its methyl signal by comparison with the peak of betaine.

### Data processing

The ^1^H NMR spectra were directly exported to AMIX 3.8 software (Bruker Biospin GmbH, Karlshure, Germany) and divided into 0.005 ppm-width buckets leading to 1874 variables. In order to remove the effects of possible variations in the water suppression efficiency, the region between 4.70 and 5.20 ppm was discarded as well as the signals of propylene glycol (3.86–3.89, 3.52–3.57, 3.42–3.46 and 1.09–1.17 ppm), an anaesthetic component. Four spectra were excluded due to baseline effects of imperfect water saturation effects. Therefore, after the NMR analysis, fifty-six spectra (6 pairs for controls; 8 pairs + 4 independent for HFS group; 12 pairs for HFS + C group) were available for multivariate analysis. To prevent problems associated to integral normalization with respect to the total area, especially when a treatment causes large changes in the spectral profile, the probabilistic quotient normalization (PQN) method was used instead^80^. To this purpose, NMR datasets (X matrix, 56 samples x 1874 buckets) were exported to MATLAB v7.4 software (The MathWorks Inc., Natick, Massachusetts, United States) and normalized on the basis of the most probable quotient of the target and a reference spectrum.

### Statistical analyses

Metabolomics (NMR) and lipidomics (GC/MS) datasets, each normalized as described above, were combined in a unique X-matrix (56 observations x 1896 buckets) and then subjected to statistical analysis using the software SIMCA-P 13 (Umetrics, Umeå, Sweden). The relative quantification of the fatty acids was replicated in the X-matrix in order to match the duplication of the NMR spectra. The results of the biochemical analysis and the FA composition were expressed as means and their Standard Error of the Mean (SEM). We used the Kolmogorov-Smirnov test to check whether the variables were normally distributed. Comparison of data between the groups was performed by one-way analysis of ANOVA or the Kruskal-Wallis test, depending on whether the distribution of data was Gaussian or not. When statistically significant, these tests were followed by a multiple comparison (*post hoc*), Tukey-Kramer or Steel-Dwass test. Statistical analysis of the data was obtained with the Sigma Stat software version 3.11 (Systat Software Inc, San Jose, Calif.). A significant difference was defined as p < 0.05.

In addition to univariate tests, multivariate statistical analyses were used. As the principal component analysis (PCA) did not produce a clear discrimination between the 3 groups, a supervised orthogonal partial least squares discriminant analysis (OPLS-DA)^81^ was applied to the X-matrix described above, in which we defined a Y-matrix as the matrix of sample classes, i.e. 0 for the controls, 1 for HFS group and 2 for HFS + C group. OPLS-DA models were calculated with Pareto scaling and the goodness-of-fit for these models estimated by R2Y, which represents the explained variance of the Y matrix, and Q2Y the predictive ability of the model. Model validation was performed by re-sampling the model 999 times under the null hypothesis, that is to say generating models with a randomly permuted Y matrix and by the use of a CV-ANOVA from SIMCA-P 13 (analysis of variance in the cross-validated residuals of a Y variable)^82^. Unpaired Student’s t-test (if the distribution was normal) or a nonparametric Mann–Whitney U test (if the distribution was non normal) were carried out on the metabolites that contributed to the discrimination between the groups through OPLS-DA models constructed from the correlation coefficients. A significant difference was defined by p < 0.05. In addition, VIP (Variable Importance for the Projection) values of metabolites were assigned by using SIMCA-P 13 where a VIP value larger than 1 indicated significant contribution to the model.

### Data Availability

The datasets generated during and/or analyzed during the current study are available from the corresponding author on reasonable request.

## Electronic supplementary material

Supplementary Information
