# Supplementary material for: Hepatic metabolic effects of Curcuma longa extract supplement in high-fructose and saturated fat fed rats
Source: Sci Rep. 2017 Jul 19;7:5880. doi: 10.1038/s41598-017-06220-0 (PMC5517472; doi:10.1038/s41598-017-06220-0)
Supplement: Supplementary file 1 — Supplementary Information [file 41598_2017_6220_MOESM1_ESM.pdf]

Hepatic metabolic effects of Curcuma longa extract supplement in high-fructose and saturated fat fed rats

Fabrice Tranchida[1]*, Zo Rakotoniaina[1], Laetitia Shintu[1], Léopold Tchiakpe[2], Valérie Deyris[1], Mehdi Yemloul[1], Pierre Stocker[3], Nicolas Vidal[3,4], Odile Rimet[2], Abel Hiol[5], Stefano Caldarelli[1]*

1 Aix Marseille Université, Centrale Marseille CNRS, iSm2 UMR 7313, 13397 Marseille, France

2 Aix-Marseille Université, Laboratoire de Nutrition-Dietétique, Faculté de Pharmacie, Marseille, France

3 Aix-Marseille Université, CNRS, Institut de Chimie Radicalaire UMR 7273, Equipe Sondes Moléculaires en Biologie et Stress Oxydant, Marseille, France

4 SARL YELEN, Ensuès la Redonne, France

5 Centre de coopération internationale en recherche agronomique pour le développement (CIRAD), UMR QualiSud, Université de La Réunion, Ecole Supérieure d'Ingénieurs Réunion Océan Indien (ESIROI), Saint Denis, France,

Corresponding Author

Fabrice Tranchida, Stefano Caldarelli

* Aix Marseille Université, Centrale Marseille CNRS, iSm2 UMR 7313, 13397 Marseille, France, e-mail: fabrice.tranchida@univ-amu.fr; s.caldarelli@univ-amu.fr. Tel: +33 491282895, Fax: +33 491289187

Supplementary Information

Table S1. Results of serum biochemical analysis and body weight after 10 weeks of diet.

**Group**	**Controls**	**HFS**	**HFS+C**
**Body Weight** (g)	435.17 ± 20.74	438.42 ± 31.85	444.75 ± 50.78
**Gain Weight** (g)	245.16 ± 8.47	248.42 ± 9.19	254.75 ± 14.66
**Relative liver weight**	0.0262 ± 0.00078	0.0310 ± 0.0010**	0.0334 ± 0.0016**
**HOMA-IR**	1 ± 0.33	10.79 ± 1.89**	6.97 ± 2.05*
**Insulin** (µg/l)	0.48 ± 0.13	3.34 ± 0.50*	2.22 ± 0.69*
**Glucose serum** (g/l)	1.07 ± 0.185	1.82 ± 0.41*	1.80 ± 0.44*
**Total cholesterol serum** (g/l)	0.60 ± 0.02	0.68 ± 0.04*	0.66 ± 0.06
**Triglycerides serum** (g/l)	0.435 ± 0.15	0.878 ± 0.22*	1.114 ± 0.36*

HFS high fructose and saturated fatty acids, C curcuma (administration of hydroalcoholic extract of tumeric 100 mg/kg/day). Relative liver weight is defined as liver weight divided by body weight. Values are mean ± S.E.M (n = 6-12 rats/group). *$P < 0.05$ vs. the control, **$P < 0.01$ vs. the control

Results previously reported for this model[5]. The body weight of HFS, HFS+C and control rats after 10 weeks of diet was similar, while a significant increase of relative liver weight was observed in both HFS groups indicating hepatomegaly as a response to a HFS diet. Compared to control, HFS diets were associated with a significant increase of serum glucose, triglycerides, cholesterol and insulin levels and HOMA-IR levels.

Table S2. Relative fatty acid composition (% of total) and estimated desaturase activities in liver of rats fed control, HFS and HFS+C diets

Fatty acid	Control	HFS	HFS+C
**C12:0 Lauric acid**	0.09 ± 0.09	nd	0.11 ± 0.04 $^{\eta}$
**C12:1n-7 Lauroleic**	0.68 ± 0.16	nd	0.15 ± 0.04 $^{\beta\,\eta}$
**C14:0 Myristic acid**	0.14 ± 0.09	0.05 ± 0.02	0.08 ± 0.02
**C14:1n-5 Myristoleic acid**	0.08 ± 0.08	0.01 ± 0.006	0.01 ± 0.003 $^{\alpha}$
**C16:0 Palmitic acid**	15.81 ± 0.53	16.88 ± 1.19	13.94 ± 0.72 $^{\eta}$
**C16:1n-7 Palmitoleic acid**	0.78 ± 0.15	1.86 ± 0.29 $^{\beta}$	1.21 ± 0.20 $^{\eta}$
**C18:0 Stearic acid**	16.6 ± 1.00	20.68 ± 1.74 $^{\alpha}$	20.97 ± 1.07 $^{\alpha}$
**C18:1n-9 Oleic acid**	6.71 ± 0.64	16.76 ± 1.12 $^{\alpha}$	16.97 ± 1.39 $^{\alpha}$
**C18:1n-7 *cis*-Vaccenic acid**	2.76 ± 0.22	4.03 ± 0.41 $^{\beta}$	3.45 ± 0.26
**C18:2n-6 Linoleic acid**	23.72 ± 1.49	12.28 ± 0.61 $^{\alpha}$	10.57 ± 0.36 $^{\alpha\,\eta}$
**C18:3n-6 gamma Linolenic acid**	0.45 ± 0.09	0.03 ± 0.02 $^{\alpha}$	0.14 ± 0.03 $^{\alpha\,\eta}$
**C18:3n-3 alpha Linolenic acid**	0.39 ± 0.06	0.02 ± 0.01 $^{\beta}$	0.03 ± 0.01 $^{\beta}$
**C20:0 Arachidic acid**	0.23 ± 0.11	0.01 ± 0.01 $^{\beta}$	0.08 ± 0.02 $^{\beta\,\eta}$
**C20:1n-9 Gondoic acid**	0.49 ± 0.12	0.07 ± 0.05 $^{\beta}$	0.22 ± 0.03 $^{\beta\,\eta}$
**C20:3n-6 Dihomo gamma linolenic acid**	0.45 ± 0.08	1.31 ± 0.15 $^{\alpha}$	1.16 ± 0.09 $^{\alpha}$
**C20:4n-6 Arachidonic acid**	19.94 ± 1.34	20.44 ± 1.57	23.3 ± 0.77 $^{\beta\,\eta}$
**C20:5n-3 Eicosopentaenoic acid**	3.15 ± 0.74	0.36 ± 0.02 $^{\beta}$	0.68 ± 0.14 $^{\beta}$
**C22:1n-9 Erucic acid**	0.36 ± 0.24	nd	nd
**C22:4n-6 Adrenic acid**	0.30 ± 0.21	0.23 ± 0.07	1.12 ± 0.53 $^{\eta}$
**C22:5n-6 Docosapentaenoic acid**	0.90 ± 0.33	0.10 ± 0.07 $^{\alpha}$	0.67 ± 0.13 $^{\varepsilon}$
**C22:6n-3 Docosahexaenoic acid**	4.87 ± 0.87	4.74 ± 0.49	4.84 ± 0.26
**C24:0 Lignoceric acid**	1.07 ± 0.27	0.12 ± 0.06 $^{\beta}$	0.26 ± 0.03 $^{\beta}$
**∑ SFA**	33.72 ± 0.84	37.74 ± 1.54	35.39 ± 0.80
**∑ MUFA**	11.78 ± 0.59	22.72 ± 0.65 $^{\beta}$	22.01 ± 1.09 $^{\beta}$
**∑ PUFA**	54.18 ± 0.91	39.52 ± 1.58 $^{\beta}$	42.51 ± 0.52 $^{\beta}$
**∑ PUFA n-3**	8.41 ± 0.21	5.13 ± 0.38 $^{\beta}$	5.56 ± 0.18 $^{\beta}$
**∑ PUFA n-6**	45.77 ± 1.01	34.4 ± 1.41 $^{\beta}$	36.95 ± 0.54 $^{\beta}$
**PUFA/MUFA**	4.68 ± 0.23	1.78 ± 0.09 $^{\beta}$	2.06 ± 0.12 $^{\beta}$
**Estimated desaturase activities**			
**Δ 9 C16:1n-7/C16:0**	0.05 ± 0.005	0.11 ± 0.014 $^{\beta}$	0.09 ± 0.009
**Δ 9 C18:1n-9/C18:0**	0.40 ± 0.04	0.81 ± 0.06 $^{\beta}$	0.81 ± 0.08 $^{\beta}$
**Δ 6 C20:3n-6/C18:2n-6**	0.02 ± 0.002	0.11 ± 0.009 $^{\beta}$	0.11 ± 0.005 $^{\beta}$
**Δ 5 C20:4n-6/C20:3n-6**	43.96 ± 4.76	15.58 ± 2.05 $^{\beta}$	20.12 ± 1.37 $^{\beta}$

Values are mean ± S.E.M (n = 6–12 rats/group). Samples were measured in duplicate. Σ SFA total saturated fatty acids, Σ PUFA total polyunsaturated fatty acids, Σ MUFA total monounsaturated fatty acids, Δ estimated desaturase activity, nd not detected.

$^{\alpha}$ significantly different from control group (p-value < 0.01)

$^{\beta}$ significantly different from control group (p-value < 0.05)

$^{\eta}$ significantly different from HFS group (p-value < 0.01)

$^{\varepsilon}$ significantly different from HFS group (p-value < 0.05)

Table S3 Assignments of metabolites ^{1}H and ^{13}C NMR

compound	^{1}H chemical shift (in ppm)	multiplicity	^{13}C chemical shift (in ppm)	assignment
Acetate	1.91	s	24.3	CH_3
Alanine	3.77	q	53.7	CH
	1.47	d	18.9	CH_3
Alpha-glucose	5.23	d	95.0	H1
	3.83	unresolved	63.4	half CH_2-C6
	3.82	unresolved	74.3	H5
	3.70	unresolved	72.7	H3
	3.53	unresolved	74.4	H2
	3.41	unresolved	78.6	H4
ATP/ADP/AMP	8.50	broad		H2-ring adenine
Beta-glucose	4.64	d	98.8	H1
	3.90	unresolved	63.6	half CH_2-C6
	3.72	unresolved	63.4	half CH_2-C6
	3.47	unresolved	78.6	H5
	3.40	unresolved	72.5	H4
	3.24	unresolved	77.0	H2
Beta-hydroxybutyrate	4.14	unresolved		betaCH
	2.39	m	49.2	half alphaCH_2
	2.30	m	49.2	half alphaCH_2
	1.19	d	24.4	gammaCH_3
Betaine	3.26	s	56.3	CH_3
	3.89	s		CH_2
Choline	4.06	m	58.0	alphaCH_2
	3.51	m	69.5	betaCH_2
	3.19	s		CH_3
Citrate	2.68	d	47.3	CH_2(i)
	2.53	d	47.3	CH_2(ii)
Creatine	3.93	s		CH_3
	3.04	s	39.5	CH_2
Lipids	5.32	broad	132.8	**CH**=CH
	2.79	broad	26.1	CH=CH**CH**2
	2.23	broad	34.2	CH2**CH**$_2$CO
	2.07	broad		CH_2
	2.01	broad		CH2**CH**$_2$C=C
	1.58	broad	25.0	**CH**2CH2CO
	1.30	broad		(CH2)n
	0.93	broad	20.9	CH_3CH_2
	0.89	broad	25.6	**CH**$_3$$CH_2$
Glutamate	2.34	m	33.9	gammaCH_2
	2.03	m	29.7	betaCH_2
Glutamine	3.74		57.6	alphaCH
	2.44	m	34.1	gammaCH_2
	2.12	m	27.1	betaCH_2
Glycerol	3.87	m	72.6	C2H
	3.63	dd	65.3	half CH2
	3.56	dd	65.3	half CH2
Glycine	3.55	s	44.8	CH_2
Glycogen	5.40	unresolved	100.3	CH1
	3.95	unresolved	73.9	CH2-6
	3.85	unresolved	63.2	
	3.79	unresolved	63.2	
	3.64	unresolved	74.3	
	3.60	unresolved	74.5	
	3.43	unresolved	72.1	
Glycoproteins (N-acetyl)	2.04	s - broad	25.0	$NHCOCH_3$
Glutathion	2.55	unresolved	34.5	gammaCH2
Histidine	7.78	s		H2
	7.03	s		H5
Isoleucine	1.97	broad		betaCH_2

	1.47<br>1.00<br>0.93	broad<br>d<br>unresolved	 17.3 	half gammaCH_2<br>gammaCH_3<br>deltaCH_3
Lactate	4.11<br>1.32	q<br>d	71.5<br>22.9	CH_3<br>CH
Leucine	3.67<br>1.71<br><br>0.94	dd<br>m<br><br>d	56.3<br>42.7<br><br>20.9	alphaCH<br>CH_2 and<br>gammaCH<br>delta-CH_3
Lysine	3.73<br>3.03<br>1.89<br>1.70<br>1.47	unresolved<br>m<br>m<br>m<br>m	57.3<br>39.2<br>33.1<br>29.6<br>25.4	alphaCH<br>epsilonCH_3<br>betaCH_2<br>deltaCH_2<br>gammaCH_2
Lysophosphatidylcholine	4.12	unresolved		C1 **CH2**OC
Methanol	3.35	s	52.0	CH_3
Methionine	2.63<br>2.12	m<br>s	31.7<br>16.8	SCH_2<br>betaCH_2
Myo-inositol	4.08<br>3.61	unresolved<br>unresolved	77.8<br>72.8	H2<br>H4, H6
Phosphatidylethanolamine	3.13	broad		Alkyl-phosphoester
Phosphocholine/<br>Glycerophosphocholine	4.32<br>3.67<br>3.21	m<br>m<br>s	62.7<br>68.3<br>56.8	alphaCH_2<br>betaCH_2<br>CH_3
Proline	3.33<br>2.34<br>2.06	unresolved<br>unresolved<br>unresolved	45.3<br>33.9<br>29.7	Half deltaCH_2<br>Half beta-CH_2<br>Half beta-CH_2
Propylene glycol	3.87<br>3.53<br>3.43<br>1.13	m<br>dd<br>dd<br>d	70.8<br>69.8<br>69.6<br>21.6	CH<br>CH_2(i)<br>CH_2(ii)<br>CH_3
Pyruvate	2.36	s	29.2	CH_3
Threonine	4.21<br>3.59	m<br>d	68.6<br>63.1	betaCH<br>alphaCH
Valine	3.58<br>2.27<br>1.03<br>0.98	unresolved<br>m<br>d<br>d	63.1<br>31.1<br>20.9<br>18.5	alphaCH_2<br>betaCH<br>Gamma-CH_3<br>Gamma′-CH_3

s = singlet; d = doublet; dd = double doublet; q = quartet; m = multiplet.

Table S4 Assignments of the polar metabolites from the Curcuma extract 1H and 13C NMR

Metabolite	^{1}H chemical shift (in ppm)	Multiplicity	^{13}C chemical shift (in ppm)	Assignment	Concentration (mM)
Acetate	1.91	s	24.3	CH_3	2.93
Alanine	3.77<br>1.49	q<br>d	53.7<br>19.3	CH<br>CH_3	0.94
Alpha-glucose	5.19<br>3.83<br>3.82<br>3.70<br>3.52<br>3.41	d<br>unresolved<br>unresolved<br>unresolved<br>dd<br>unresolved	95.6<br>63.4<br>74.3<br>72.7<br>74.4<br>78.6	H1<br>half CH_2-C6<br>H5<br>H3<br>H2<br>H4	5.58
Aspartate	3.89<br>2.80<br>2.67	dd<br>dd<br>dd	55.3<br>39.7<br>39.0	CH_2<br>CH_2<br>CH	0.89
4-Aminobutyrate	3.01<br>2.30<br>1.89	t<br>unresolved<br>unresolved	41.8<br>37.4<br>26.6	gammaCH_2<br>alphaCH_2<br>betaCH_2	0.33
Beta-glucose	4.59<br>3.89<br>3.80<br>3.72<br>3.44<br>3.40<br>3.20	d<br>unresolved<br>unresolved<br>unresolved<br>t<br>unresolved<br>unresolved	99.2<br>63.6<br>63.6<br>63.4<br>78.6<br>72.5<br>77.0	H1<br>half CH_2-C6<br>half CH_2-C6<br>half CH_2-C6<br>H5<br>H4<br>H2	7.49
Betaine	3.28<br>3.89	s<br>s	56.3	CH_3<br>CH_2	0.22
Choline	4.06<br>3.51<br>3.22	m<br>m<br>s	58.0<br>69.5<br>56.9	alphaCH_2<br>betaCH_2<br>CH_3	1.39
Citrate	2.68<br>2.53	d<br>d	46.3	CH_2(i)<br>CH_2(ii)	1.42
Ferulate	7.32<br>7.22<br>7.10<br>6.92<br>6.36<br>3.90	d<br>d<br>dd<br>d<br>d<br>s	143.6<br>113.9<br>124.3<br>118.6<br>125.6<br>58.9	CH=CH<br>CH=CH<br>CH=CH<br>CH=CH<br>CH=CH<br>CH_3	0.58
Fructose	4.09<br>4.08<br>3.92<br>3.55<br>3.54	d<br>d<br>m<br>d<br>d	78.4<br>77.4<br>72.2<br>65.6<br>65.6	H2<br>H3<br>H1<br>H5<br>H5	7.97
Formate	8.47	s	173.5	CH	1.68
Fumarate	6.53	s	138.3	CH=CH	0.049
Glutamine	3.74<br>2.42<br>2.10	unresolved<br>unresolved<br>unresolved	58.5<br>33.5	alphaCH<br>gammaCH_2<br>betaCH_2	N.D
Glycerol	3.77<br>3.64<br>3.55	unresolved<br>unresolved<br>unresolved	75.6<br>65.1<br>65.1	C2H<br>half CH2<br>half CH2	N.D
4-Hydroxycinnamic acid	7.50<br>7.34<br>6.90<br>6.36	dd<br>dd<br>dd<br>d	131.6<br>143.9<br>118.6<br>125.1	H13, H14<br>H17<br>H15, H16<br>H18	0.49
Lactate	4.07<br>1.33	q<br>d	71.5<br>22.9	CH_3<br>CH	3.70
Malate	4.27<br>2.67<br>2.36	m<br>dd<br>q	73.2<br>46.0<br>46.0	alphaCH<br>betaCH_2<br>betaCH_2	2.23
Malonate	3.11	s	51.0	CH_2	0.26
Nicotinate	8.97<br>8.60	unresolved<br>dd		H2<br>H6	0.08

	8.28<br>7.53	m<br>dd		H4<br>H5	
Succinate	2.42	s	37.0	CH_2	0.88
Sucrose	5.41<br>4.18<br>4.04<br>3.85<br>3.82<br>3.81<br>3.79<br>3.67<br>3.52<br>3.44	d<br>d<br>t<br>m<br>m<br>m<br>m<br>s<br>dd<br>t	95.6<br>80.2<br>77.4<br>84.5<br>74.4<br>63.0<br>65.1<br>65.0<br>74.4<br>75.6	Glc-H1<br>Fruc-H3<br>Fruc-H2<br>Fruc-H1<br>Glc-H5<br>Glc-H6<br>Fruc-H6<br>Fruc-H5<br>Glc-H2<br>Glc-H4	24.45
Tryptophan	7.72<br>7.52<br>7.31<br>7.26<br>7.21	unresolved<br>d<br>s<br>unresolved<br>unresolved	119.6<br>113.8<br>127.0<br>124.6<br>122.6	H7<br>H6<br>H2<br>H9<br>H8	0.70
Tyrosine	7.19<br>6.87	unresolved<br>d	133.2<br>118.4	Ar-CH<br>Ar-CH	0.38
Uracil	7.53<br>5.77	d<br>unresolved			0.47
Valine	2.26<br>1.06<br>1.01	m<br>d<br>d	31.1<br>20.9<br>18.5	betaCH<br>Gamma-CH_3<br>Gamma′-CH_3	0.58
U1	7.11<br>2.28	s<br>s	132.2<br>51.1		
U2	3.33	m	51.5		
U3	6.26<br>2.10	m<br>s	126.8<br>37.0		
U4	6.14<br>1.97	s<br>s	126.8<br>23.2		
U5	9.73	s			
U6	9.69	s	156.7		

s = singlet; d = doublet; dd = double doublet; q = quartet; m = multiplet, U unknown

Table S5.Composition of the diets and fatty acid profile

Constituents (g/100 g dry weight)	Control diet	HFS diet
Protein	19	19
Methionine	0.3	0.3
Starch	62	−
Sucrose	3	−
Cellulose	5	−
Fructose	−	61.7
Minerals and vitamins	7	7
Choline	0.04	0.04
Lard	−	12
Soybean and fish lipid sources	3.5	−
Main fatty acids (% total fatty acids)		
16:0	15.5	24.6
18:0	traces	13.8
16:1n-7	2.3	2.2
18:1n-9	25	37.9
18:2n-6	48	10.8
18:3n-3	0.5	0.8
∑SFA	17	40.6
∑MUFA	30	46.9
∑PUFA	53	12.5

HFS high fructose and saturated fatty acids, ∑ SFA total saturated fatty acids, ∑ MUFA total monounsatured fatty acids, ∑ PUFA total polyunsatured fatty acids.

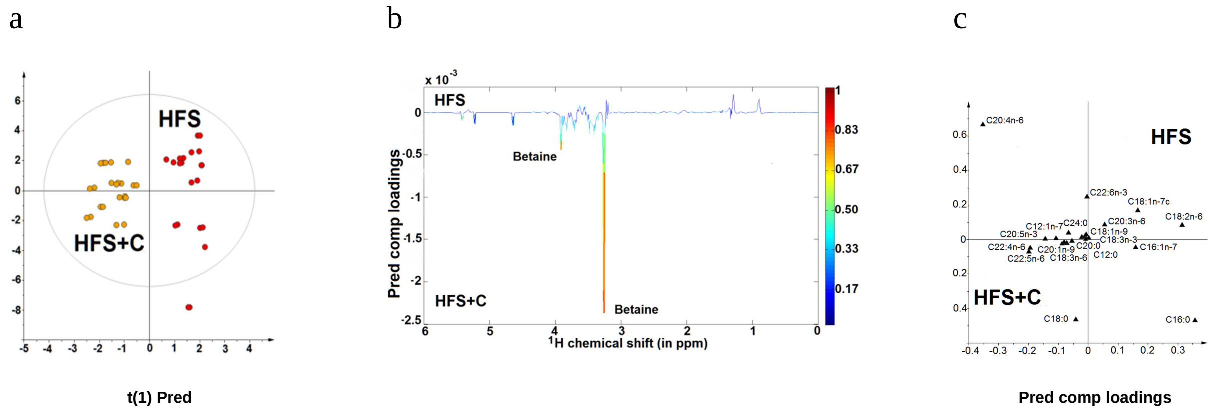

Figure S1 OPLS-DA score (a) and loadings plots representing the weights of the NMR signals (b) and the relative fatty acid contents (c), along the predictive component derived from OPLS-DA model of liver samples obtained from HFS and HFS+C groups.

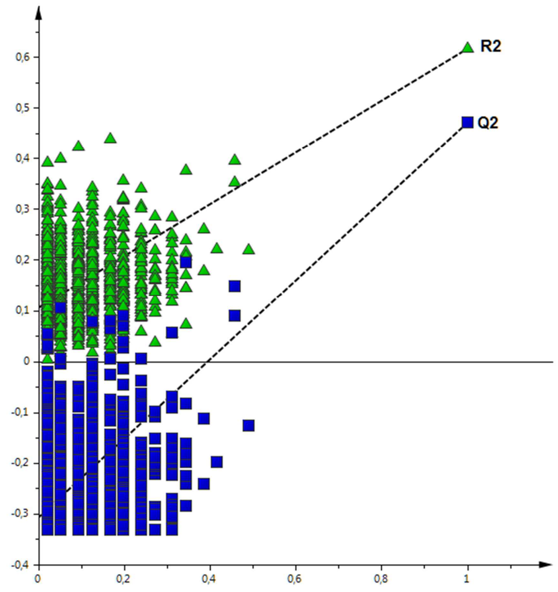

Figure S2 Permutation test plot for the OPLS-DA model (number of permutations, 999; intercepts: R2 = 0.0, 0.09; Q2 = 0.0, -0.31).

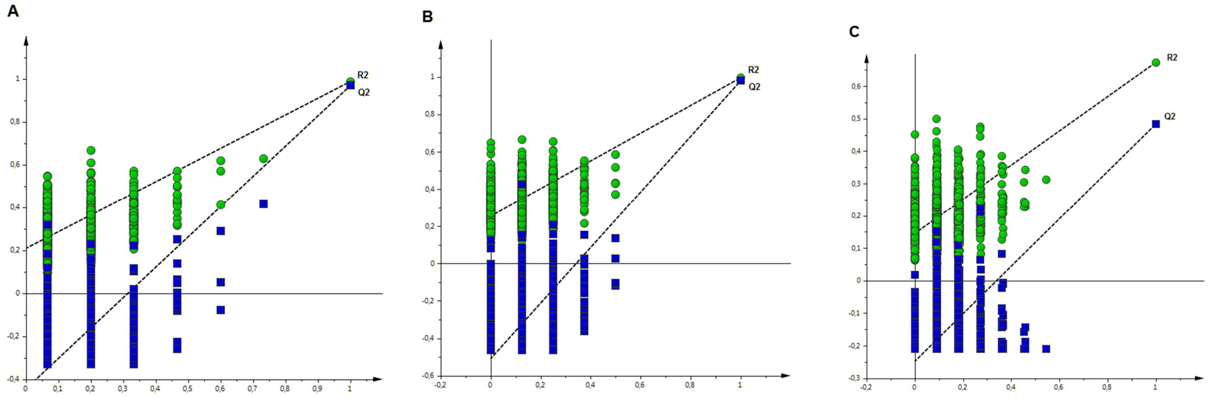

Figure S3 The permutation test plots for the OPLS-DA models for classification of (A) control and HFS groups (intercepts: R2=0.0, 0.209; Q2=0.0, -0.444), (B) control and HFS+C groups (intercepts: R2=0.0, 0.255, Q2=0.0, -0.509), and (C) HFS and HFS+C groups (intercepts: R2=0.0, 0.146; Q2=0.0, -0.248) (number of permutations, 999).

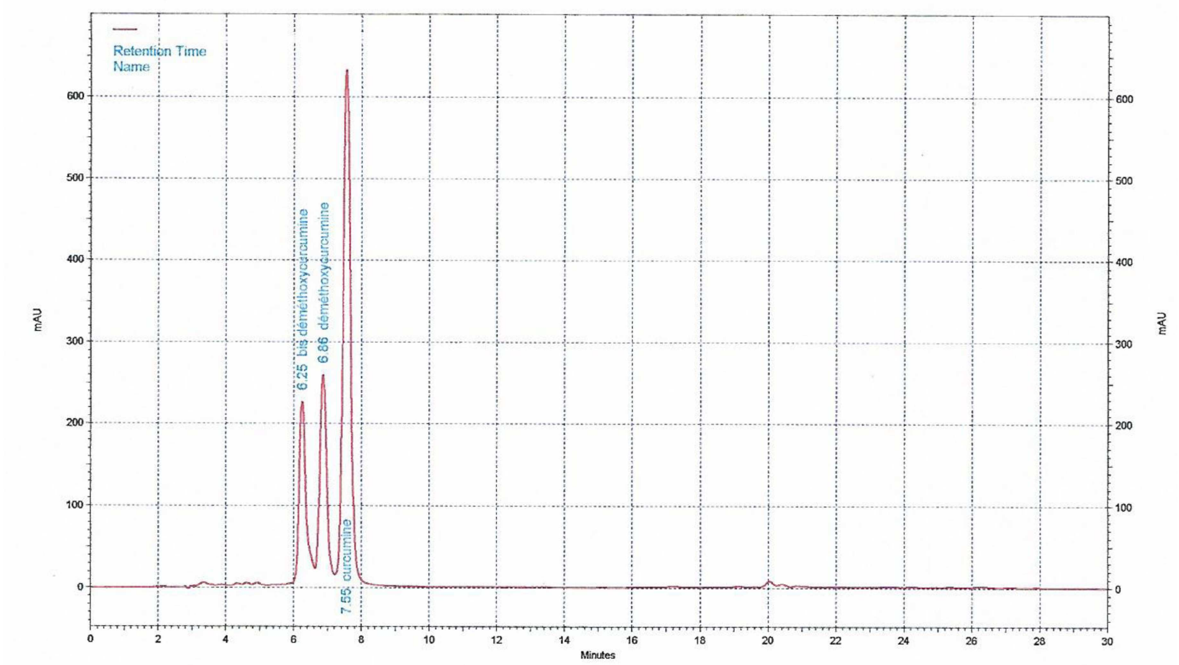

Name	Retention Time (min)	Area	Area %	Sim
bis demethoxycurcumin	6.25	3188986	18.97	0.999
demethoxycurcumin	6.86	3730670	22.20	1.000
curcumin	7.55	9886775	58.83	1.000

Figure S4 HPLC chromatogram of curcuminoid compounds in the Curcuma extract

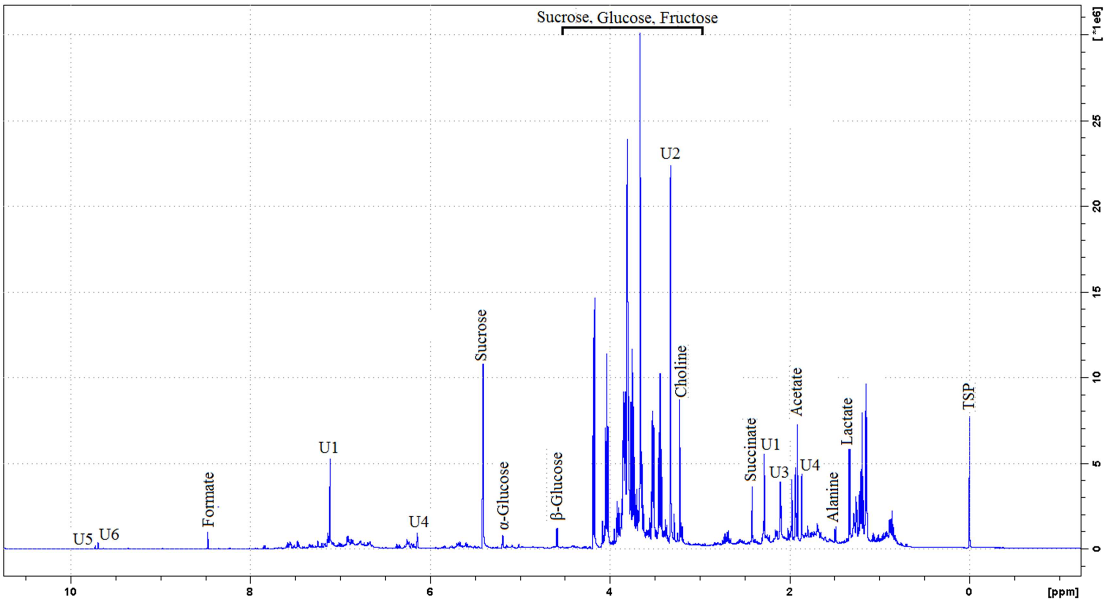

Figure S5 Representative ^{1}H NMR spectra of Curcuma extract (the major identified metabolites are shown).

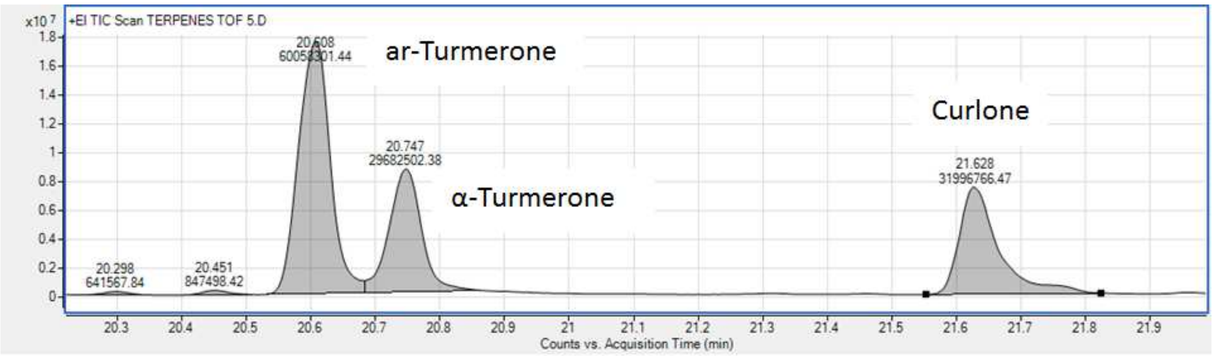

Name	Retention Time (min)	Area	Area %
ar-Turmerone	20.608	60058301.44	49.34
α-Turmerone	20.747	29682502.38	24.38
Curlone	21.628	31996766.47	26.28

Figure S6 GC/QTOF MS chromatographic profile of identified non-polar metabolites from the hexane extract of Curcuma extract

A) **ar-Turmerone**

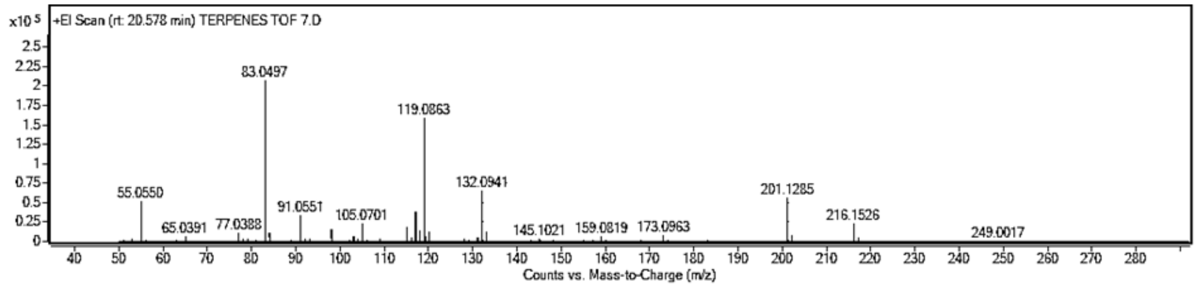

B) **α-Turmerone**

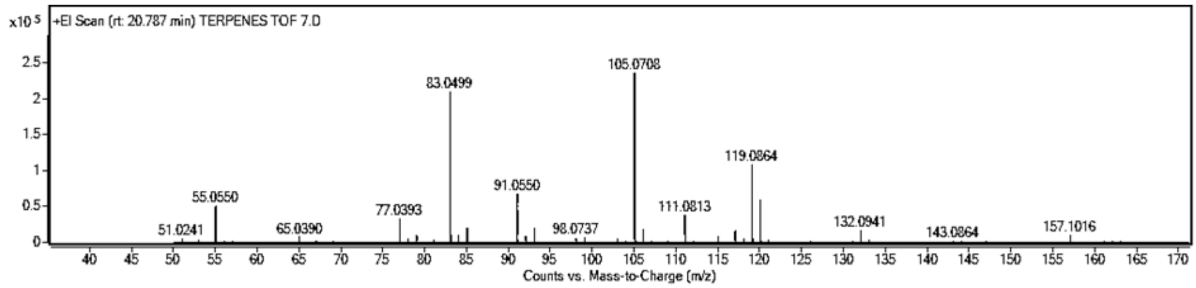

C) **Curlone**

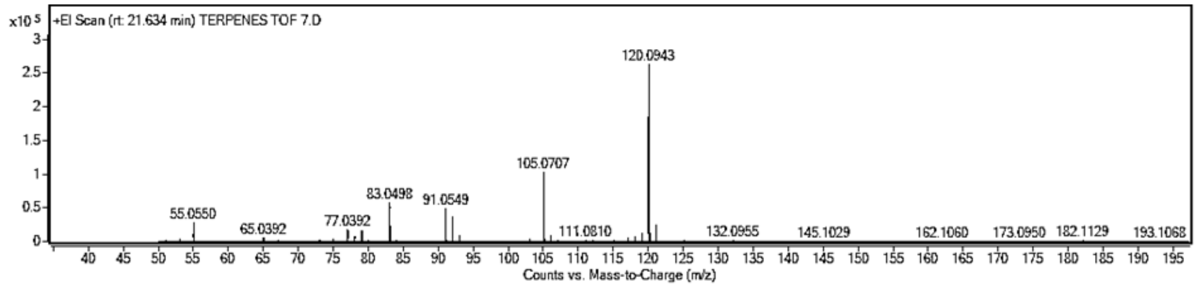

Retention Time (min)	Identified metabolite	Formula	Accurate Mass	Main fragments m/z (% bp)[a]
20.60	ar-Turmerone	$C_{15}H_{20}O$	216.1514	83.0497 (100), 119,0863 (80.6), 132.0941 (32.9)
20.78	α-Turmerone	$C_{15}H_{22}O$	218.1671	105.0708 (100), 83.0499 (88.2), 119.0864 (47.3)
21.63	Curlone	$C_{15}H_{22}O$	218.1671	120.0943 (100), 105.0707 (38.4), 83.0498 (21.8)

[a]m/z, relative intensity to base peak (% bp)

Figure S7 mass spectra of terpenes detected from the hexane extract of Curcuma extract. (A) ar-Turmerone, (B) α-Turmerone, (C) Curlone.

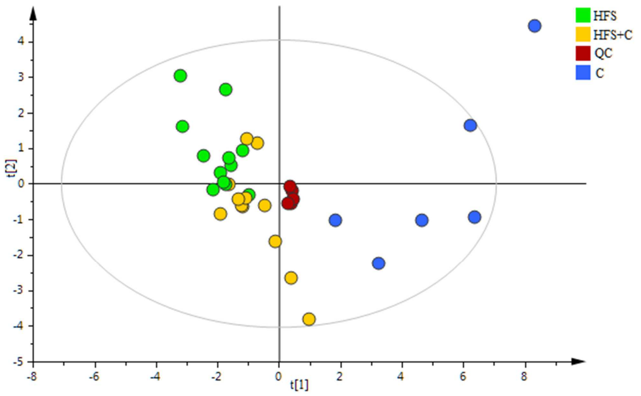

Figure S8 PCA score plot of FAs from the QCs and the study samples

Data S1 NMR analysis of polar extract

- Procedures for preparation of sample for NMR analysis

For the extraction of polar metabolites, 600 μL of methanol (d4), 400 μL of phosphate buffer solution (1.9 mM Na_2HPO_4, 8.1 mM NaH_2PO_4, 150 mM NaCl, 1 mM 3-(trimethylsilyl)propionic-2,2,3,3-d4 acid (TSP), pH 7.4) containing 90:10 D_2O/H_2O (v/v) were added to 50 mg of dry hydroalcoholic extract of *Curcuma longa*, vortexed for 1 min and sonicated for 20 min at 40 °C. The mixture was centrifuged at 17 000 g for 10 min, and then the supernatants (600 μL) were transferred into 5 mm NMR tubes. Methanol (d4) and TSP provided a field frequency lock and a chemical shift reference (^{1}H, δ 0.00), respectively.

- Instrumental analysis of polar extract by NMR analysis

All NMR experiments were carried out on a Bruker Avance III spectrometer operating at 600 MHz for the ^{1}H frequency equipped with a 5 mm BBFO probe with z-gradient. Spectra were acquired at 300 K. A pulse-acquire sequence with water signal presaturation zgpr during a relaxation delay of 2 s was recorded. Two hundred fifty-six transients, an acquisition time of 4.00 s and 64k data points were collected using a spectral width of 8000 Hz. A 0.5 Hz line-broadening function was applied to all spectra prior to Fourier transformation (FT). Subsequently, the spectra were phased and baseline corrected manually and referenced to the TSP methyl signal (δ = 0.00 ppm). Assignments of the metabolite signals were performed using ^{1}H−^{1}H TOCSY, ^{1}H−^{13}C HSQC, ^{1}H−^{13}C HMBC spectra and using reference signals published in the literature. Metabolites were identified using Chenomx Profiler, a module of Chenomx NMR Suite version 7.6, combined with analyses of 2D NMR experiments. The reference signal (TSP) corresponding to a known concentration was used to determine the concentration of individual compounds.

Data S2 GC/QTOF MS analysis of non-polar extract

- Procedures for preparation of sample for GC/TOF MS analysis

To extract non-polar metabolites, dried hydroalcoholic extract of *Curcuma longa* (50 mg) was transferred into 8 mL glass vials and dissolved in methanol-water (2 mL, 1:1, v/v), and then the sample was vortexed for 1 min. After addition of n-hexane (2 mL), the mixture was vortexed twice for 1 min each. The combined extracts were then placed at room temperature for 20 min, with the two phases separated by centrifugation at 2500 g for 5 min at 4 °C. The supernatant (non-polar phase) was transferred into a new glass vial.

- Instrumental analysis of non-polar extract by GC/TOF MS

Accurate mass measurements were carried out in full scan mode with an Agilent 7890B/7200 GC/QTOF System (Agilent Technologies,Parc Technopolis - ZA Courtaboeuf, Les Ulis, France). A cross-linked 5% phenyl-methylpolysiloxane (Agilent; HP-5MS ultra inert) (30 m×0.25 mm, 0.25 μm film thickness) capillary column was employed. Samples (1 µl) were performed with an injector operating in split mode (1:10) set at 270°C and the oven temperature began at 60°C for 2 min and programmed from 60 to 100°C at 20°C/min, then to 220°C at 4°C/min, and then to 300°C at 25°C/min. The pressure of the carrier gas (Helium) was maintained at 0.69×105 Pa until the end of the temperature program. Instrument temperatures were 280°C for transfer line and 230°C for the ion source. Accurate mass spectra were recorded across the range m/z 50–700 at 4 GHz. The QTOF-MS instrument provided atypical resolution ranging from 8009 to 12252 from m/z 68.9955 to 501.9706. Perfluorotributylamine (PFTBA) was utilized for daily MS calibration. The GC/QTOF MS raw data were analyzed using the MarkerLynx Applications Manager version 4.1 (Waters) for mass spectral peak identification. Compounds were identified by comparing mass spectra with the National Institute of Standards and Technology mass spectral library (National Institute of Standards and Technology (NIST) MS search 2.0).
